# Interrater Agreement of Physicians Identifying Lung Sliding Artifact on B-Mode And M-Mode Point of Care Ultrasound (POCUS)

**DOI:** 10.24908/pocusj.v10i01.17807

**Published:** 2025-04-15

**Authors:** Ross Prager, Hans Clausdorff Fiedler, Delaney Smith, Derek Wu, Robert Arntfield

**Affiliations:** 1 Division of Critical Care Medicine, Western University, London, ON, CAN; 2 Sección de Medicina de Urgencia. Pontificia Universidad Católica de Chile, CHL; 3 Faculty of Mathematics, University of Waterloo, Waterloo, ON, CAN; 4 Schulich School of Medicine and Dentistry, Western University, London, ON, CAN; 5 Division of Critical Care Medicine, Western University, London, ON, CAN

**Keywords:** point of care ultrasound, lung ultrasonography, reliability, POCUS, pneumothorax

## Abstract

**Background:**

Chest point of care ultrasound (POCUS) is a first-line diagnostic test to identify lung sliding, an important artifact to diagnose or rule out pneumothorax. Despite enthusiastic adoption of this modality, the interrater reliability for physicians to identify lung sliding is unknown. Additionally, the relative diagnostic performance of physicians interpreting B-mode and M-mode ultrasound is unclear. We sought to determine the interrater reliability of physicians to detect lung sliding on B-mode and M-mode POCUS.

**Methods:**

We performed a cross-sectional interrater agreement study surveying acute care physicians on their interpretation of 20 B-mode and M-mode POCUS clips. Two experienced clinicians determined the reference standard diagnosis. Respondents reported their interpretation of each POCUS B-mode clip or M-mode image. The primary outcome was the interrater agreement, determined by an intra-class correlation coefficient (ICC).

**Results:**

From September to November 2023, there were 20 survey respondents. Fourteen (70%) respondents were resident physicians. Respondents were confident or very confident in their skill performing chest POCUS in 14 (70%) cases, with 19 (90%) performing chest POCUS every week or more frequently. The ICC on B-mode was 0.44 and for M-mode was 0.43, indicating moderate agreement. There were no significant differences in interrater reliability between subgroups of confidence or experience.

**Conclusion:**

There is only moderate interrater reliability between clinicians to diagnose lung sliding. Clinicians have superior accuracy on B-mode compared to M-mode clips.

## Background

Chest point of care ultrasound (POCUS) is a safe and portable tool to diagnose pulmonary pathology at the bedside, including pneumothorax (PTX) [1–6]. The diagnosis of PTX on chest POCUS is primarily made through the identification of “lung sliding” artifact, a shimmering of the pleural line that is formed from contact between the two pleural layers. When air occupies the space between the pleura during a PTX, this shimmering disappears and there is absent lung sliding [7, 8]. Despite enthusiastic adoption of this modality, some studies suggest variable diagnostic accuracy, with concerns about clinicians missing a significant proportion of PTXs [9, 10]. Proposed explanations for this variability include confounding factors at a patient level (e.g. emphysema), or significant interrater reliability between operators.

To improve diagnosis, some clinicians use motion (M-mode) ultrasound to diagnose PTX. M-mode produces a time lapse image of the 2D clip at the designated line on the ultrasound screen [11]. Patients with PTX have a “barcode” sign, whereas patients with normal lung sliding have a “seashore” sign. Often, the use of M-mode ultrasound is proposed as a solution to unclear lung sliding on B-mode ultrasound, however, enthusiasm and utility of this practice is likely overstated [12].

To further understand the utility and limitations of chest POCUS to diagnose PTX, we must first explore the core diagnostic test metric of interrater reliability. Although many studies have looked at diagnostic accuracy metrics (e.g. sensitivity and specificity), there is a paucity of data on interrater reliability. Additionally, the interrater reliability of B-mode compared with M-mode POCUS is uncertain. This prospective survey study of acute care clinicians characterized the interrater reliability of clinicians assessing lung sliding for B-mode and M-mode POCUS.

## Methods

### Ethics Approval

Research ethics approval for research on human subjects was obtained from Western Research Ethics Board (Approval#: 122668). Completion of the survey constituted implied consent.

### Study Design

We conducted a cross-sectional interrater agreement study of acute care physicians to determine the interrater reliability and diagnostic accuracy of chest POCUS to detect absent lung sliding artifact.

### Setting and Respondents

A convenience sample of residents or attending physicians at London Health Sciences Centre (LHSC), a large tertiary hospital system with established acute care ultrasound fellowship programs, were emailed with an invitation to participate. Physicians with experience using chest POCUS for PTX rule-out from the following acute care specialties were eligible to participate: emergency medicine, anesthesia, internal medicine, or intensive care. Members of the research team were excluded from participating.

### Survey Design

The survey consisted of two sections: demographics and interpretation. In the demographics section, the following characteristics were obtained: age, sex, post graduate year (PGY), specialty, current use of chest POCUS and self-reported confidence with chest POCUS use. In the interpretation section, clinicians interpreted 40 chest POCUS clips (20 B-mode videos and 20 M-mode images) obtained from 20 patients. The survey is provided in Appendix 1.

The B-mode and M-mode chest POCUS clips were downloaded from the Qpath (Telexy Inc.) database, a cloud database archiving chest POCUS images at our institution. All clips were obtained using a phased array probe (any vendor), with the abdominal imaging preset. A convenience sample of clips from adult patients with suspected PTX from December 13th, 2022, to June 6th, 2023, were chosen. They were selected to have approximately equal lung sliding present and lung sliding absent clips. To determine the reference standard diagnosis for the presence or absence of lung sliding, two chest POCUS experts with fellowship training in ultrasound (RP and HCF) independently interpreted the clip, with a third expert reviewer (RA) used for disagreements. All clips were anonymized for any patient health information prior to export and were downloaded in MP4 format. The images were then uploaded to REDCap electronic data capture tool.

### Image Interpretation

Prior to starting the survey, respondents were given written instructions with definitions for lung sliding and lung point, and images showing barcode sign and seashore sign (Appendix 2). During the survey, respondents were asked to provide a diagnosis of lung sliding present or lung sliding absent for each clip. Respondents were asked to label a lung sliding clip as “absent lung sliding” if lung sliding was absent from any position along the pleural line (to capture lung point artifact). Respondents were asked to label “lung sliding present” if there was shimmering of the pleural line throughout the entire rib interspace, which included the presence of lung pulse.

### Primary Outcome

The primary outcome was the interrater agreement (measured by ICC) of respondents in their interpretation of lung sliding, stratified between B-mode and M-mode POCUS.

### Secondary Outcomes

The secondary outcomes were the diagnostic accuracy for B-mode and M-mode to detect absent lung sliding (the disease present class given that clinically it could signify PTX).

### Subgroup Analyses

Pre-specified subgroup analyses for ICC were performed based on provider experience, use, and self-reported confidence.

### Statistical Analysis

All data was reported as count data (n, %), means (standard deviation [SD]), or medians (interquartile ranges [IQR]) as appropriate. Interrater reliability was determined by calculating an ICC for the B-mode and M-mode clips separately. Interrater reliability was compared between subgroups by examining the 95% confidence interval (CI).

Diagnostic accuracy – including sensitivity, specificity, precision, and accuracy – was calculated for B-mode and M-mode separately. Accuracy metrics for B-mode and M-mode were compared using the Mann-Whitney U test.

### Sample Size Calculation

*A priori*, we performed a sample size calculation to calculate the number of readers and number of clips required to obtain our expected ICC of 0.5 (moderate), with 95% CI of an ICC ± 0.2. [13] (Appendix 3). Allowing for a 20% missing data from the survey, we required 20 B-mode and 20 M-mode clips to be read by 20 independent readers.

## Results

### Surveyor Characteristics

Of the 35 surveys sent, there were 20 respondents. All respondents completed the B-mode interpretation, but one respondent did not complete the M-mode because the images did not display properly on their device. Twelve (60%) respondents were male, with a mean age of 30.7 years (SD 3.6). The distribution between residents, attendings, and PGY are provided in Table 1. Sixteen (80%) of respondents reported they were comfortable or very comfortable with chest POCUS, with ten (50%) respondents reporting more than three years of experience with chest POCUS. Eighteen (90%) reported using POCUS on a weekly basis or more frequently. All (100%) respondents had additional POCUS training outside of residency.

**Table 1.** Respondent demographics and expertise with lung ultrasound

**Demographics**	
Age, mean (SD)	30.7 (3.6)
Male Sex, n (%)	12 (60%)
**Post Graduate Year, n (%)**	
Year 3	1 (5%)
Year 4	5 (25%)
Year 5	4 (20%)
Year 6	5 (25%)
Year 7	4 (20%)
Year 8	1 (5%)
**Medical Background, n (%)**	
Resident	14 (70%)
Attending	6 (30%)
**POCUS Expertise**	
**Years of Experience using POCUS, n (%)**	
Less than 1 year	2 (10%)
1 to 3 years	8 (40%)
3 to 5 years	7 (35%)
5 to 10 years	3 (15%)
**Frequency of POCUS use in their Practice, n (%)**	
Every month	2 (10%)
Every week	9 (45%)
Almost every day	5 (25%)
Every day	4 (20%)
**Additional POCUS Training, besides Residency, n (%)**	
Ultrasound Fellowship	3 (15%)
POCUS Elective Rotation	16 (80%)
Live POCUS Course	9 (45%)
Online POCUS Course	4 (20%)
**Applications for which they use LUS n (%)**	
Pneumothorax / Lung Sliding Assessment	20 (100%)
Pleural Effusion Assessment / Thoracocentesis guidance	19 (95%)
Interstitial Syndrome Assessment (A vs B Lines)	15 (75%)
Consolidation Assessment (Pneumonia / Atelectasis)	16 (80%)
**Self-Reported Confidence with LUS, n (%)**	
Somewhat comfortable	4 (20%)
Comfortable	8 (40%)
Very Comfortable	8 (40%)
**Routinary use of M-Mode for Pneumothorax Assessment, n (%)**	
Yes	17 (85%)
No	3 (15%)

### Interrater Agreement

**Figure 1.**
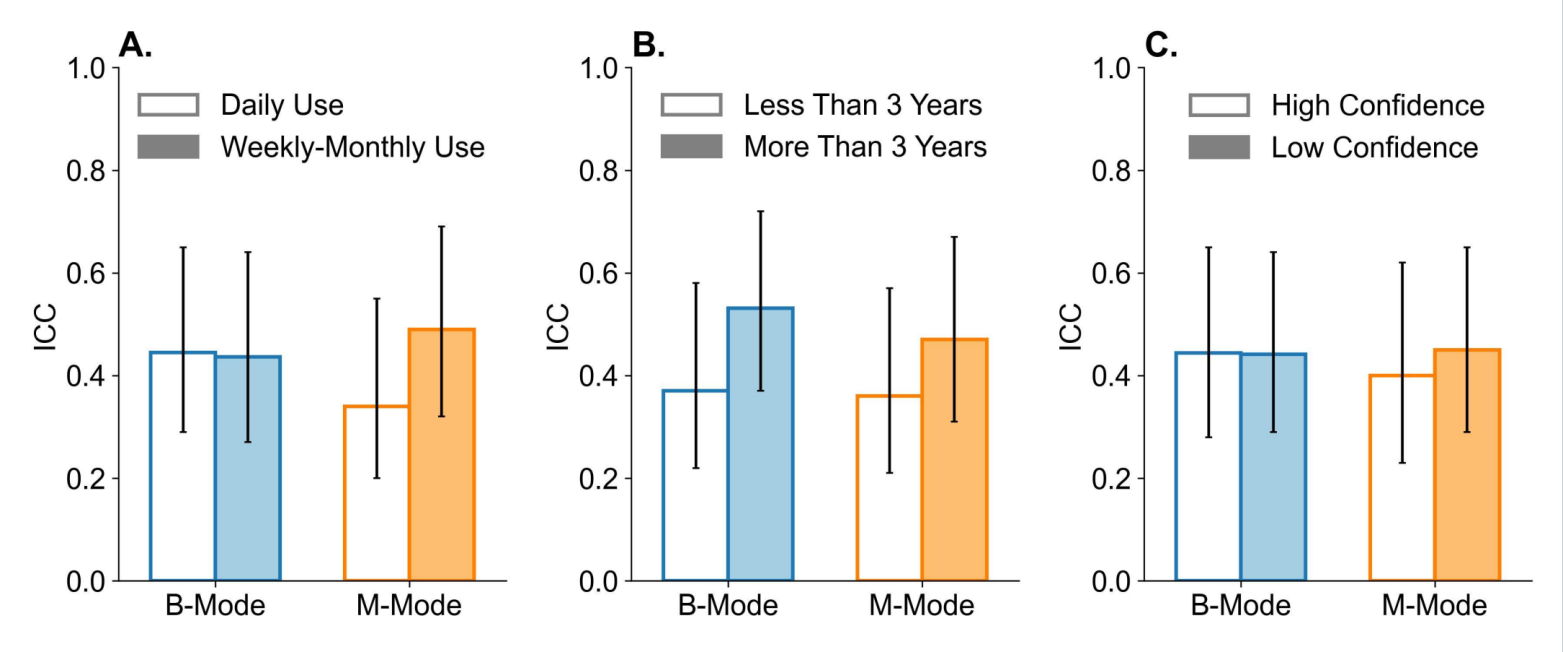
ICC for the interpretation of lung sliding on B-mode (blue) and M-mode (orange) POCUS, stratified by interpreter self-reported use of POCUS (A), experience with POCUS (B), and confidence with chest POCUS (C). Error bars reflect the 95% CI.

The ICC for B-mode (N = 20) was 0.44 (95% CI 0.30 – 0.64), and the ICC for M-mode (N = 19) was 0.43 (95% CI 0.29 – 0.63). Figure 1 summarizes the difference in ICC across various subgroups of interest, including the surveyors self-reported frequency of POCUS use (Panel A), years of POCUS experience (Panel B), and confidence with chest POCUS (Panel C). The CIs suggest no significant difference in ICC for either type of chest POCUS.

### Diagnostic Accuracy

**Figure 2.**
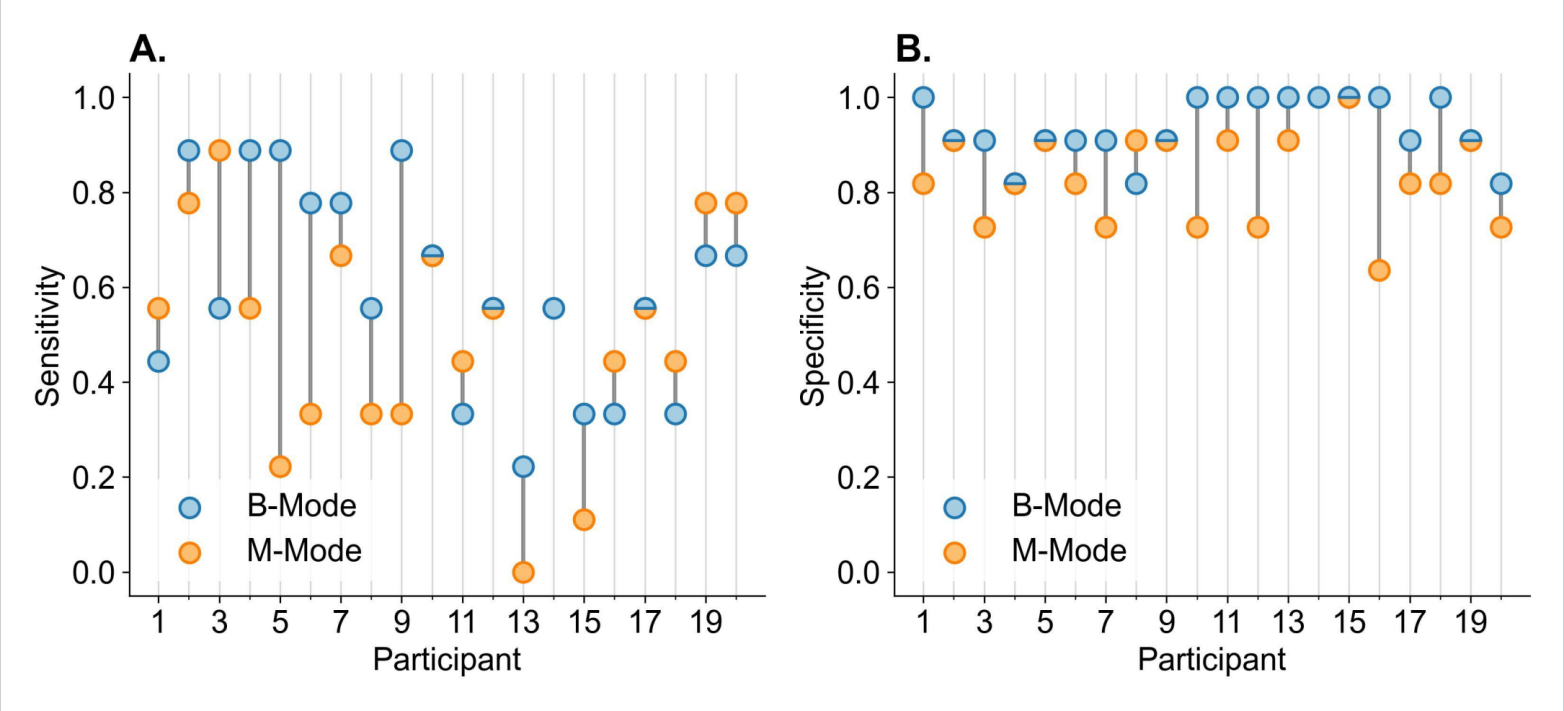
Respondent diagnostic accuracy on B-mode (blue markers) and M-mode (orange markers) POCUS, as evaluated by the sensitivity (A) and specificity (B) for the detection of absent lung sliding. Participant 14 did not interpret M-mode.

On B-mode, the sensitivity to detect absent lung sliding was 0.59 (95% CI 0.50 to 0.68), with a specificity of 0.94 (95% CI 0.91 to 0.97). The low sensitivity means clinicians were more likely to misclassify a true absent lung sliding as having lung sliding (false negative). Of note, there was significant variation in sensitivity across respondents. (Figure 2)

The interpretation of M-mode was more challenging than B-mode, with M-mode respondents yielding a mean sensitivity of 0.50 (95% CI 0.40 to 0.60) and mean specificity of 0.83 (95% CI 0.79 to 0.87). Twelve (63%) respondents had lower sensitivity (Figure 2A) for M-mode compared to B-mode, and 18 (95%) had lower specificity (figure 2B) for M-mode compared to B-mode. The overall accuracy estimates are provided in Table 2.

**Table 2.** Inter-rater agreement (ICC) and mean (SD) diagnostic accuracy of surveyors for the detection of absent lung sliding on B-Mode and M-Mode ultrasound.

	ICC	Diagnostic Accuracy			
		Sensitivity	Specificity*	Accuracy*	Precision*
**B-Mode**	0.44	0.59 (0.21)	0.94 (0.06)	0.78 (0.08)	0.91 (0.09)
**M-Mode**	0.43	0.50 (0.23)	0.83 (0.09)	0.68 (0.09)	0.68 (0.19)
Asterisks (*) indicate metrics whose underlying distributions were found to be statistically different for B-mode and M-mode POCUS using a Mann-Whitney U test.					

**Figure 3.**
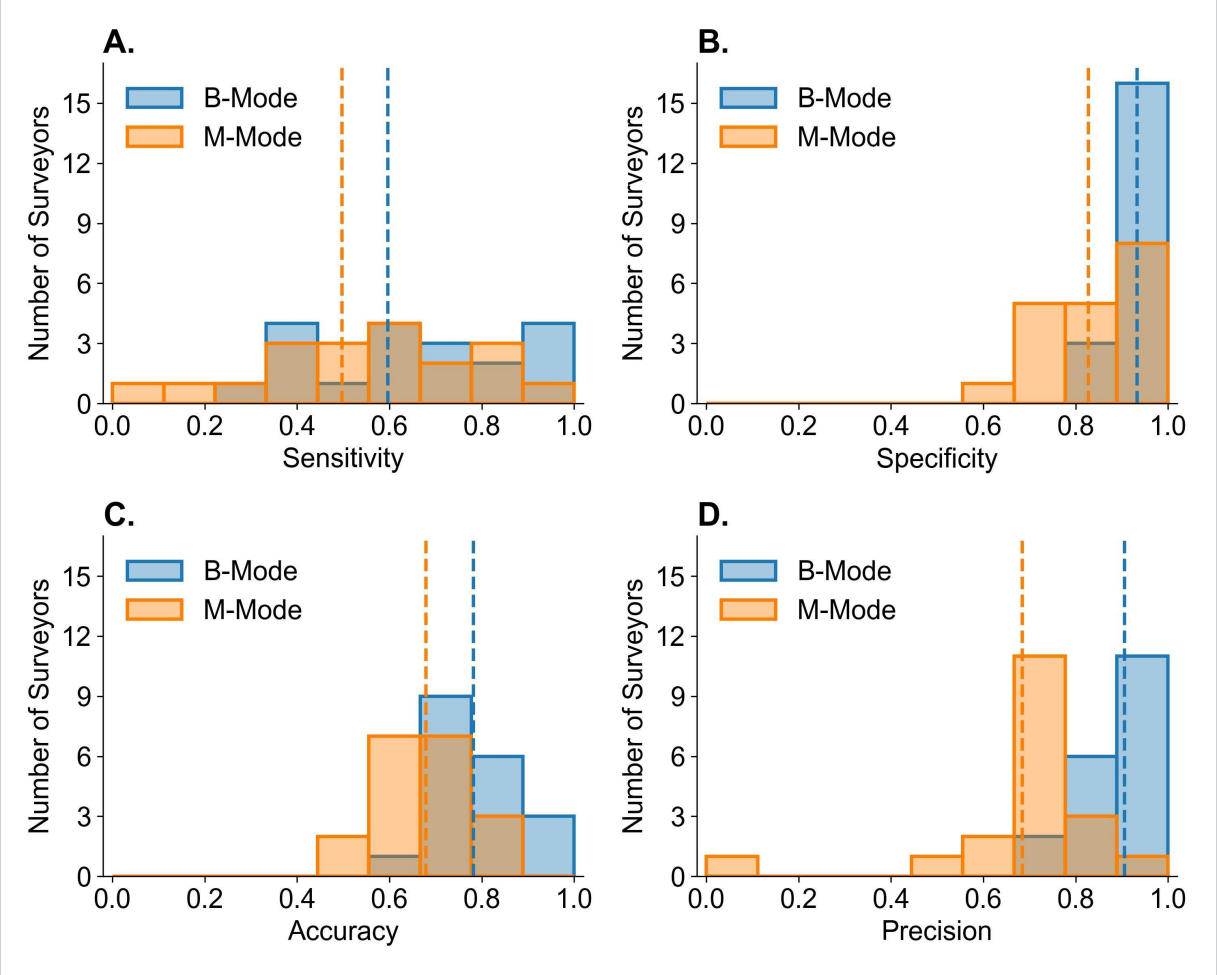
Comparing the distribution of surveyor diagnostic accuracy metrics between B-mode (blue) and M-mode (orange) POCUS. Dashed lines indicate the mean of the distribution. Except for sensitivity (A), the distributions of surveyor specificity (B), accuracy (C), and precision (D) differed significantly between ultrasound modes using a Mann-Whitney U test at a significance level of 0.05.

When pooled, M-mode had similar sensitivity (mean difference [MD] -0.09 (P=0.24)), lower specificity (MD -0.11, p<0.001), lower accuracy (MD -0.11, p=0.001), and lower precision (MD -0.23, p<0.001) compared to B-mode (Figure 3).

Appendix 4 summarizes the differences in mean sensitivity and specificity across subgroups of interest. There were no significant differences between groups.

## Discussions

In this interrater reliability study, physicians had moderate agreement for the detection of lung sliding artifact on B-mode and M-mode POCUS. Overall, clinicians had lower sensitivity (ability to identify absent lung sliding) than specificity. Additionally, clinicians had higher specificity, accuracy, and precision for B-mode compared to M-mode. Accuracy estimates did not vary based on ultrasound use frequency, experience, or self-reported confidence.

A combination of technological improvements (increased portability) and integration of ultrasound into medical education [14–18] have contributed to gradual but steady adoption of chest POCUS into clinical practice. The use of chest POCUS to rule out PTX – a serious diagnosis that clinicians often fear missing – has been a particularly attractive use. Unfortunately, with only moderate interrater reliability for both B-mode and M-mode, widespread adoption requires consideration. Questions surrounding the reliability of chest POCUS to diagnose PTX have previously been raised in the literature, owing to significant variability in diagnostic accuracy for chest POCUS to detect PTX [9, 10]. One potential explanation for low interrater reliability is that the training of clinicians is inadequate – however, our cohort consisted of clinicians with significant experience in chest POCUS. Almost all physicians routinely used chest POCUS in practice and had additional training in chest POCUS use beyond residency. Alternatively, there is a slightly inconvenient narrative that it is easy to identify lung sliding when it is present (high specificity), but people intrinsically find it more difficult to identify the absence of lung sliding motion (poor sensitivity). This is mirrored in the radiology literature, where positive pathology can be identified quickly, but subtle findings or identifying the absence of a finding can be more difficult [19–21].

Our study also refutes the mindset that M-mode can serve as an adjudicator in cases where B-mode findings are equivocal. In fact, M-mode had lower diagnostic accuracy on multiple metrics compared with B-mode POCUS. A previous study showed that for more novice operators (who have performed less than 250 scans), M-mode may offer modest diagnostic improvement to B-mode for lung sliding [12]. However, this is unlikely to overcome the limitations in interrater reliability we have seen in this study [12]. Interestingly, where people have difficulty with M-mode POCUS, machine learning algorithms using M-mode are quite successful, as M-mode's barcode-like output can be readily identified by computers [7].

While widespread and accurate integration of chest POCUS into clinical practice is valuable, interrater reliability may be the greatest barrier to scaling this solution across healthcare systems. Although training is often proposed to overcome this, even among experienced providers this may still be a limiting factor. Further work into understanding the learning curves among providers with different backgrounds for chest POCUS would be beneficial. Artificial intelligence has increasingly become an attractive approach to scale the reach and impact of point-of-care imaging devices. In the lung sliding space, preliminary in-silico (computer) models for lung sliding have high diagnostic accuracy, however, their application to the bedside or in real-world scenarios are an area of ongoing investigation [7].

## Limitations

There are several notable limitations of this study. First, a survey study of chest POCUS does not perfectly reflect the application of chest POCUS at the bedside, which has acquisition and interpretation. Additionally, during clinical application of chest POCUS, providers can obtain additional views in cases of uncertain lung sliding. Furthermore, although respondents represented an experienced cohort of clinicians in terms of training and frequency of use of chest POCUS, most were early in their careers. With that said, we believe our cohort reflects a high standard of POCUS proficiency. Because the cohort was a single centre is also a limitation, and training and experience at different centers could impact interrater reliability metrics. Finally, some missing data (one user's M-modes did not display) reduces our power; however, drop-out was accounted for in our power calculations.

## Conclusions

Clinicians have only moderate interrater reliability interpreting lung sliding artifact on chest POCUS, which was similar between B-mode and M-mode. Overall, the diagnostic accuracy for B-mode was higher than for M-mode, with higher specificity, accuracy, and precision. Finally, operator experience, confidence, and frequency of chest POCUS use did not significantly impact diagnostic accuracy.









## Past Presentation

No previous presentations.
