# Supplementary material for: Interrater Agreement of Physicians Identifying Lung Sliding Artifact on B-Mode And M-Mode Point of Care Ultrasound (POCUS)
Source: POCUS J. 2025 Apr 15;10(1):92–8. doi: 10.24908/pocusj.v10i01.17807 (PMC12057470; doi:10.24908/pocusj.v10i01.17807)
Supplement: Supplementary file 1 [file pocusj-10-01-17807-s001.pdf]

Appendix 1. Copy of the demographic part and interpretation part of the survey

**What's your age?**	Text
**What's your sex?**	Male/Female/Other
**In which Postgraduate year (PGY) are you? (if in practice indicate years since graduating medical school)**	Text
**What's your current practice status?**	Medical Student<br>Resident<br>Emergency Medicine Attending<br>Internal Medicine Attending<br>Critical Care Attending<br>Anesthesia Attending<br>Attending (other) ________
**Select your experience using POCUS in your practice**	< 1 year<br>1-3 years<br>3-5 years<br>5-10 years<br>> 10 years
**How often do you use POCUS as part of your everyday clinical practice?**	Never<br>Every month,<br>Every week<br>Almost every day<br>Every day
**Beyond your training during your residency, What additional POCUS training have you completed. (select all that apply)**	Ultrasound Fellowship<br>Specific POCUS Elective Rotation<br>Live POCUS Course<br>Online POCUS Course<br>None of the above
**For what applications do you use lung Ultrasound?**	Pneumothorax / Lung Sliding Assessment<br>Pleural Effusion Assessment / Thoracocentesis guidance<br>Interstitial Syndrome (Differentiating A vs B pattern)<br>Consolidation (Pneumonia vs Atelectasis)<br>Other
**How would you describe your confidence using lung Ultrasound?**	Not Comfortable<br>Somewhat Comfortable<br>Comfortable<br>Very Comfortable
**Do you routinely use M-Mode (in addition to 2D/B Mode) in the assessment of pneumothorax?**	Yes<br>No
