# Supplementary material for: Interrater Agreement of Physicians Identifying Lung Sliding Artifact on B-Mode And M-Mode Point of Care Ultrasound (POCUS)
Source: POCUS J. 2025 Apr 15;10(1):92–8. doi: 10.24908/pocusj.v10i01.17807 (PMC12057470; doi:10.24908/pocusj.v10i01.17807)

Appendix 2. Instructions to respondents prior to completion of the survey

Dear Participant:

On behalf of the whole investigation team, we would like to thank you for your effort and time. Your participation in this survey will contribute to our understanding of how accurately individuals can diagnose the presence or absence of lung sliding on Lung Ultrasound Clips and in M-Mode images.

This survey consists of 40 questions each involving the interpretation of a 3 second lung ultrasound clip or an M-Mode. Its completion time is estimated to be 20 minutes. Please carefully follow the instructions provided for each question.

General Instructions: Make sure you have a stable internet connection and are in a quiet environment to focus on the survey. This survey is made to be accessed through a mobile device (like a cellphone or tablet) or through a PC. Take your time to carefully review each question and provide your responses as accurately as possible.

Question Format: For each B-mode, please carefully observe the motion of the lung surface and determine if "lung sliding" is present using the definitions below. Once complete, please indicate your level of confidence using the provided scale. For each M-mode, make the same binary determination using the M-Mode image provided. The B-mode and M-modes are not linked together and are in a random order.

Definitions on B-mode

Lung sliding PRESENT refers to examples where the lung sliding or lung pulse artifacts are present throughout the duration of the clip over the entirety of the pleural line

Lung sliding ABSENT refers to examples where the lung sliding or lung pulse artifacts are not definitively present throughout the duration of the clip. Includes instances of absent lung sliding and lung point.

The definitions of lung sliding, lung pulse, and lung point are provided here for your reference.

• Lung sliding: A dynamic ultrasound artifact observed as a shimmering or sliding movement of the pleural line (interface between the lung and chest wall) during respiration. It indicates normal lung movement and excludes pneumothorax.

• Lung pulse: An artifact seen on B-mode ultrasound, where the pleural line exhibits synchronous movement with the cardiac cycle, typically at the lower border of the lung. It suggests a connection between the visceral and parietal pleura and rules out PTX at the transducer location.

• Lung point: A specific B-mode ultrasound finding observed in pneumothorax, where the pleural line transitions from sliding to static (absent sliding), revealing the point where the collapsed lung meets the chest wall. It helps diagnose pneumothorax and its extent.

Definitions on M-mode:

Lung sliding PRESENT is indicated by the presence of the 'seashore sign'. See image for reference.

Lung sliding ABSENT is indicated by the presence of the 'barcode sign'. See the image for reference.

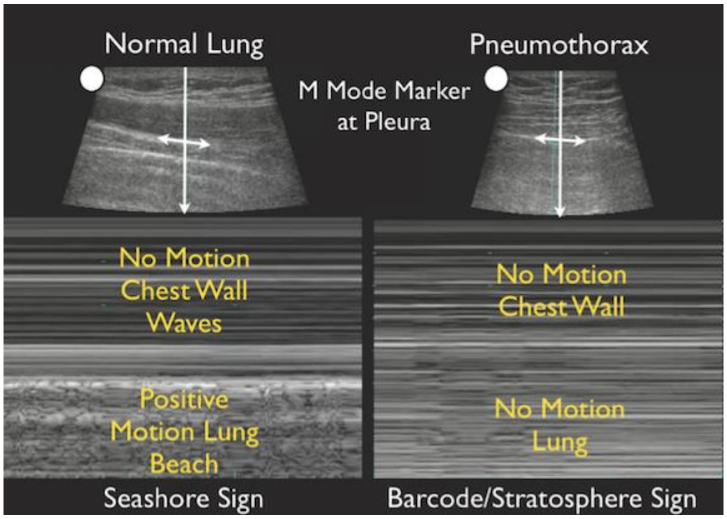

Supplement: Supplementary file 2 [file pocusj-10-01-17807-s002.pdf]
