# Supplementary material for: Interrater Agreement of Physicians Identifying Lung Sliding Artifact on B-Mode And M-Mode Point of Care Ultrasound (POCUS)
Source: POCUS J. 2025 Apr 15;10(1):92–8. doi: 10.24908/pocusj.v10i01.17807 (PMC12057470; doi:10.24908/pocusj.v10i01.17807)
Supplement: Supplementary file 3 [file pocusj-10-01-17807-s003.pdf]

Appendix 3. Sample size calculation (Bonett, 2002)

$$n = 8z_{\alpha/2}^2\{(1-\tilde{\rho}_1)^2(1+(k-1)\tilde{\rho}_1)^2\}/\{k(k-1)w^2\}+1$$
