# Supplementary material for: Interrater Agreement of Physicians Identifying Lung Sliding Artifact on B-Mode And M-Mode Point of Care Ultrasound (POCUS)
Source: POCUS J. 2025 Apr 15;10(1):92–8. doi: 10.24908/pocusj.v10i01.17807 (PMC12057470; doi:10.24908/pocusj.v10i01.17807)

Appendix 4. Mean sensitivity (row A) and specificity (row B) for the interpretation of lung sliding on B-Mode and M-Mode ultrasound, stratified by interpreter self-reported use of POCUS (column i), B: experience with POCUS (column ii), and confidence with LUS (column iii). Error bars reflect standard deviation.

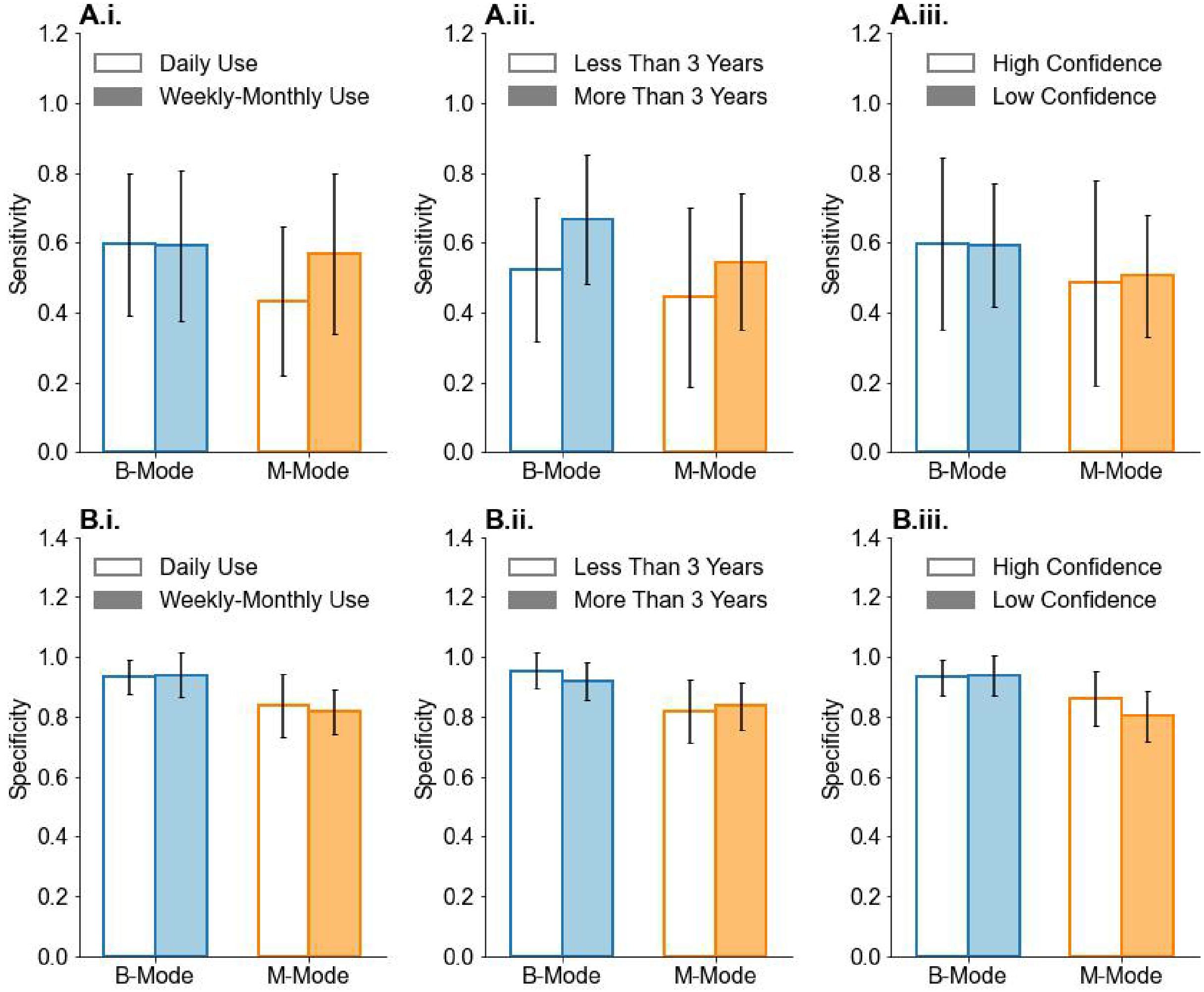

Supplement: Supplementary file 4 [file pocusj-10-01-17807-s004.pdf]
